# Selecting Clinically Relevant Gait Characteristics for Classification of Early Parkinson’s Disease: A Comprehensive Machine Learning Approach

**DOI:** 10.1038/s41598-019-53656-7

**Published:** 2019-11-21

**Authors:** Rana Zia Ur Rehman, Silvia Del Din, Yu Guan, Alison J. Yarnall, Jian Qing Shi, Lynn Rochester

**Affiliations:** 1 Institute of Neuroscience/Institute for Ageing, Newcastle University, Newcastle Upon Tyne, NE4 5PL UK; 2 School of Computing, Newcastle University, Newcastle Upon Tyne, NE4 5TG UK; 3 School of Mathematics, Statistics, and Physics, Newcastle University, Newcastle Upon Tyne, NE1 7RU UK; 4 The Newcastle Upon Tyne Hospitals NHS Foundation Trust, Newcastle Upon Tyne, NE7 7DN UK

**Keywords:** Diagnostic markers, Parkinson's disease

## Abstract

Parkinson’s disease (PD) is the second most common neurodegenerative disease; gait impairments are typical and are associated with increased fall risk and poor quality of life. Gait is potentially a useful biomarker to help discriminate PD at an early stage, however the optimal characteristics and combination are unclear. In this study, we used machine learning (ML) techniques to determine the optimal combination of gait characteristics to discriminate people with PD and healthy controls (HC). 303 participants (119 PD, 184 HC) walked continuously around a circuit for 2-minutes at a self-paced walk. Gait was quantified using an instrumented mat (GAITRite) from which 16 gait characteristics were derived and assessed. Gait characteristics were selected using different ML approaches to determine the optimal method (random forest with information gain and recursive features elimination (RFE) technique with support vector machine (SVM) and logistic regression). Five clinical gait characteristics were identified with RFE-SVM (mean step velocity, mean step length, step length variability, mean step width, and step width variability) that accurately classified PD. Model accuracy for classification of early PD ranged between 73–97% with 63–100% sensitivity and 79–94% specificity. In conclusion, we identified a subset of gait characteristics for accurate early classification of PD. These findings pave the way for a better understanding of the utility of ML techniques to support informed clinical decision-making.

## Introduction

Parkinson’s disease (PD) affects approximately 10 million people worldwide, with a doubling of the global burden over the past 25 years due to increasing longevity and longer disease duration^1^. PD has both motor and non-motor symptoms, and diagnosis is based on clinical features^2,3^. The diagnostic accuracy of clinical diagnosis of PD in differentiating PD largely from other neurological disorders is only 74% when performed by non-experts and 80% by movement disorder specialists; this is particularly problematic in the early stages of disease^4^. The Movement Disorder Society has recently proposed new clinical diagnostic criteria for PD that incorporates non-motor manifestations^5^. However, other diagnostic aids are needed to improve accuracy. Gait performance is a marker of global health in general, predicting mortality, morbidity, falls and neurodegenerative disorders^6^. Gait impairments are a common feature of PD, appearing early and evolving over time^7–10^. They could therefore inform early diagnosis^11^. Moreover, evidence suggests they are present in the prodromal phase and could identify risk of disease in the prodromal phase^12,13^ along with the possibility of different phenotypes of PD. Collectively this could lead to more personalized care and clinical trials.

Gait is typically described by its spatiotemporal characteristics such as step length, step velocity, step width, step time, swing time, stance time (mean gait characteristics) and their respective variability and asymmetry (dynamic gait characteristics)^6,14,15^. A comprehensive conceptual gait model organized these spatiotemporal gait characteristics into five domains (pace, rhythm, variability, asymmetry and postural control) based on factor analysis and highlighted its importance due to their association with clinical attributes including cognitive impairment in PD^15^. For example, factors in the pace domain may help to differentiate mild cognitive impairment from normal cognition^16^, whereas postural control may act as an early biomarker for asymmetrical neurodegenerative diseases such as PD^17^, while variability in gait predicts falls in older adults and PD^18^. Currently, gait impairment is commonly described using a univariate approach, precluding an understanding of the contribution of multiple gait characteristics. Identifying the optimal combination of gait characteristics to better define PD is therefore a priority in order to develop its use as a possible tool to aid diagnosis and management of PD^19^.

Machine learning (ML) provides a method to identify the best combination of clinically relevant spatiotemporal gait characteristics to address questions around disease classification^20,21^. Earlier work using sequential forward selection, minimum redundancy, maximum relevancy, and mutual information based methods applied to the vertical ground reaction forces has been used to find suitable statistical features for PD classification^11^. A range of other methods have also been tested for selection of suitable features in neurodegenerative diseases^22–24^. However, the feature selection method that has small searching space for optimal results is missing. As a starting point, a good feature selection technique should select the features that have a high correlation with the response variable (PD or healthy controls classes) and minimum redundancy among the gait characteristics^25^. Therefore, there is a need to identify the suitable ML modes and the optimal combination of gait characteristics for classification of PD.

Widely reported machine learning models in literature for PD classification are support vector machine, random forest, k-nearest neighbours, classification and regression trees, neural networks, and logistic regression^11,20–22,24,26–33^. However there is no consensus and studies are difficult to compare. Therefore, a comprehensive ML approach whereby previous ML models are implemented on the larger dataset with a comprehensive combination of gait characteristics is needed in order to identify the most relevant gait features for classification of PD. The choice of gait characteristic is important for the models so that their findings are easy to interpret. Based on the literature, gait characteristics vary widely, often with no consistency across studies or rationale for feature inclusion for classification of PD^11,20–22,24,26–33^. Features based upon common spatiotemporal gait characteristics that can be easily understood in relation to the underlying disease are helpful and pre-existing gait models inform comprehensive feature selection^15^. For example, asymmetry may be helpful in early PD as degeneration of dopaminergic cells occurs with an asymmetrical distribution. Other limitations of previous work include participants with more severe disease, a relatively small sample size and lack of ground truth data to quantify the best gait features. Together this reduces the generalizability, validity and applicability of results. Therefore, large studies for PD classification in people with less severe disease using a selection of gait characteristics that are easily interpretable and easily quantified are needed.

This is the largest early study in which a comprehensive set of clinically relevant spatiotemporal gait characteristics extracted from early cohort are used for classification of PD. The aims of the study are to identify: 1) suitable ML models to apply to gait features to discriminate PD and healthy controls (HC); and 2) the optimal combination of clinically relevant gait characteristics for early classification of PD. In order to achieve these aims, first we need to understand the input features (gait characteristics) in ML models as training data and then propose a ML framework for finding the optimal traditional ML models for PD classification while addressing generalizability issues.

## Methods

### Participants

303 subjects were recruited from the “Incidence of Cognitive Impairment in Cohorts with Longitudinal Evaluation-GAIT” (ICICLE-GAIT) study^15^. All the recruited subjects from ICICLE-GAIT were used for analysis without applying any additional inclusion or exclusion criterian. Among the cohort, 119 were people with early PD diagnosed according to the UK Parkinson’s Disease Brain Bank criteria^34^ by a movement disorder specialist^35^ and 184 healthy control subjects (HC). Ethical approval was obtained from the “Newcastle and North Tyneside research ethics committee” (REC No. 09/H0906/82). All subjects gave written informed consent before participating in this study. In addition, confirming that, all the methods and experiments were performed according to the declaration of Helsinki.

### Demographic and clinical measures

Participants’ demographic characteristics such as age, height, weight, and BMI were recorded. Severity of the PD motor symptoms was assessed using Hoehn and Yahr scale^36^ and part III of the modified version of Movement Disorder Society Unified Parkinson’s Disease Rating Scale (MDS-UPDRS)^37^; tremor dominant and postural instability and gait difficulty (PIGD) phenotypes were calculated from MDS-UPDRS^38^. Freezing of gait (FOG) was assessed with the new freezing of gait questionnaire^39^ and levodopa equivalent daily dose (LEDD) was also measured. Cognition was assessed with the Mini-Mental State Examination (MMSE)^40^; and balance confidence was evaluated with the balance self-confidence scale^41^.

### Testing protocol and experimental setup

**Figure 1:**
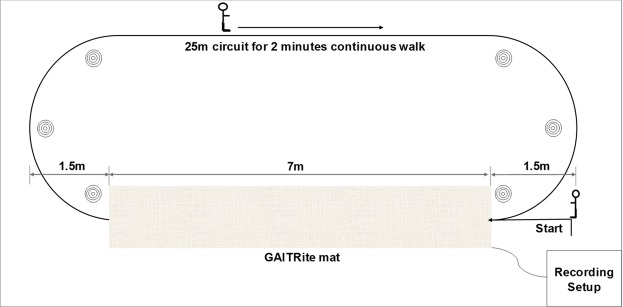
Layout of gait assessment in lab.

Participants were instructed to walk at their preferred pace continuously for 2 minutes on a 25 m oval circuit^34^, gait was repeatedly sampled as participants walked on an instrumented walkway (Platinum model GAITRite; 7.0 meters long and 0.6 meters wide) placed in the middle of the circuit (Fig. 1). GAITRite has a spatial accuracy of 1.27 cm and temporal accuracy of 1 sample (240 Hz, ~4.17 ms). PD patients were assessed whilst in a clinically defined “ON” state.

### Data processing and outcome

From GAITRite, each individual step’s data were extracted with Microsoft Access. Mean gait characteristics were calculated by taking the average of all trials, and dynamic gait characteristics were calculated according to the methods described previously^34^. In total, 16 gait characteristics were derived based on previous work and grouped to broad independent domains for easy interpretation (pace, rhythm, variability, asymmetry, and postural control)^15^.

### Statistical analysis

Independent t-tests were used to examine the difference between groups (PD vs. HC) for demographic data and gait characteristics. The area under the curve was used to check their discriminative power for classification. Pearson’s correlation between gait characteristics was also evaluated to see the independence and redundancy.

### Framework for classification modelling

**Figure 2:**
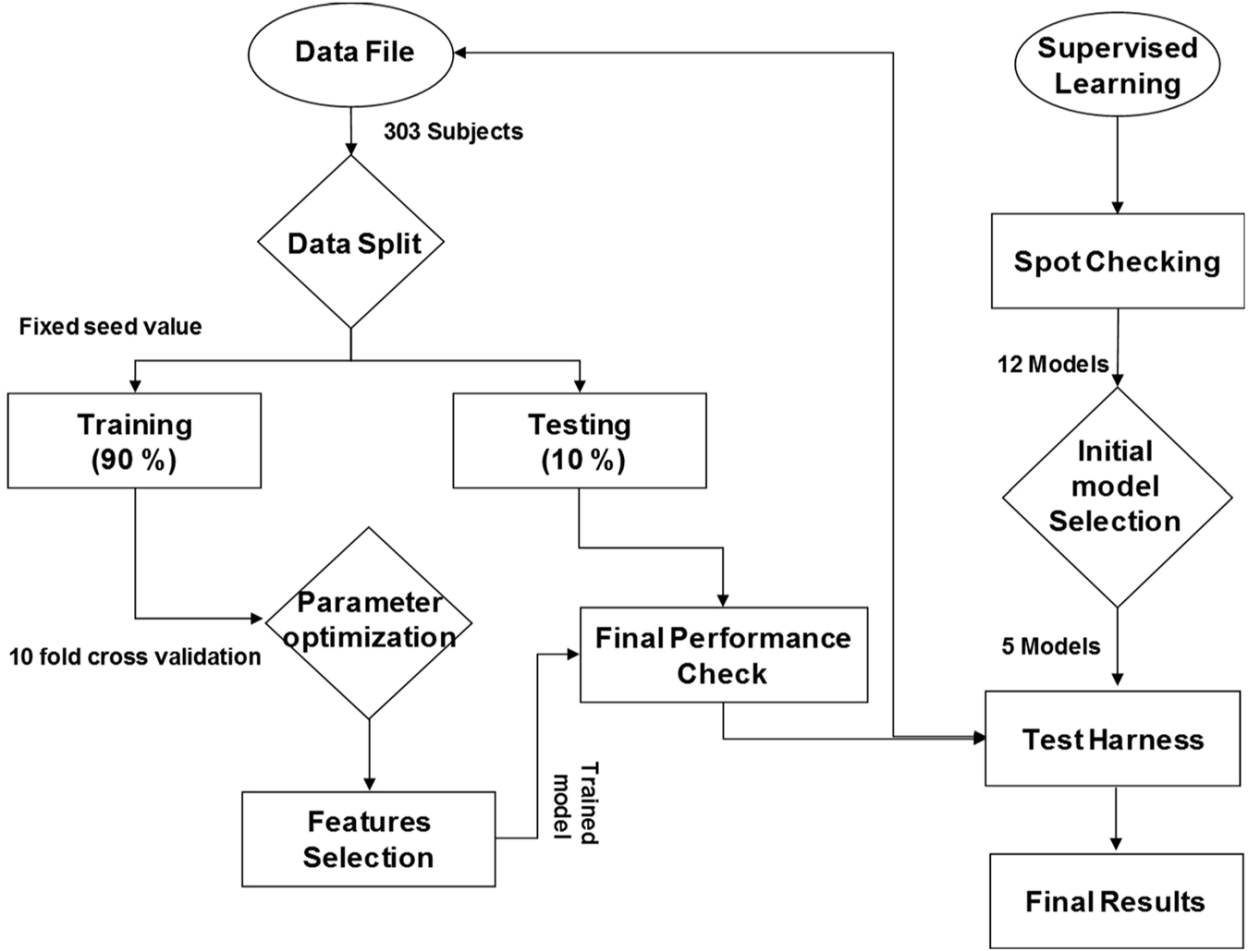
Framework for machine learning modelling for PD Classification.

For supervised ML modelling, a comprehensive approach was adopted. Different ML models such as logistic regression (LR)^32^, linear discriminant analysis (LDA)^42^, k-nearest neighbour (KNN)^43^, classification and regression tree (CART)^11^, Naive Bayes (NB)^20^, support vector machine (SVM)^21,32,33^, random forest (RF), bagged decision tree (BDT), extra tree classifier (ETC), AdaBoost classifier (AC), gradient boosting classifier (GBC)^44^, and voting methods^22^ containing LDA, NB, and SVM were employed. A ML framework was proposed for the selection and evaluation of these models with a test harness (Fig. 2).

The proposed ML framework used 16 gait characteristics as predictor variables (standardized data: zero-mean, unit variance) and disease status (PD or HC) as a response variable. Firstly, based on literature^11,20–22,32,33,42–44^, 12 widely reported linear and non-linear models in the classification of PD were selected. Secondly, based on spot checking of 12 models on the whole dataset with help of 10-fold cross-validation (CV), an initial selection of five models was made for further analysis. Then a test harness was developed for further testing of the models. As a first step, the dataset was split into training (90%) and testing (10%). This stage was important to compare the training and testing performance of the initially selected models to check the distribution of the data. Then model selection was performed again on the training and testing results. Selected models’ hyperparameters were tuned further on the training data to obtain the best classification accuracy on the validation data set. A grid search method was utilized with 10 fold cross validation to find the appropriate hyperparameters. Selection of the gait characteristics and the desired optimal number were also performed based on their contribution in the ML models. For ML modelling Python is used with standard libraries^45^.

### Gait characteristics selection techniques

Recursive feature elimination (RFE) technique with RF, linear kernel SVM, and LR was used to select the optimal number of features based on their contribution in the classification accuracy, evaluated through the 10-fold validation^46^. To further validate these results, model performance was compared using the test data set. The gait characteristics’ importance was also quantified using RFE with linear kernel SVM and LR. For RF, information gain is used to know the relative importance of the gait characteristics, which is a widely used method in bioinformatics for features selection^47,48^. The general algorithm for RFE is given below^49^ for gait characteristics selection. For implementation, standard commands from SciKit-learn library in Python were used.

### Model Inputs

Training data (n = 303 subjects)

X_0_ = [x_1_, x_2_, x_3_, … x_k_…, x_n_]^T^

Class labels (PD or HC)

y = [y_1_, y_2_, y_3_, … y_k_…, y_n_]^T^

### Initiation of selection process

Selected features, N

s = [1, 2, …, N]

Feature ranking

r = []

Recursive repetition until s = []

Restrict the training data to good features indices

X = X_0_(:,s)

Training the model

α = model-train(X, y)

Compute the weight for each gait feature in s

w = $$\sum _{k}{\alpha }_{k}{y}_{k}{X}_{k}$$

Calculation for ranking

c_i_ = (w_i_)^2^ for all i

Find the features with the smallest ranking criterion

f = argmin(c)

Update the features ranking list

r = [s(f), r]

Eliminate the features with the smallest ranking criterion

s = s(1:f−1, f + 1:length(s))

### Output

Ranked gait features list r.

## Results

Table 1 shows the demographic, cognitive and clinical characteristics of participants. In keeping with early disease, mean MDS-UPDRS III score was 25.4, and mean LEDD 175.9 mg/day. Only 11 participants had evidence of freezing of gait (FOG) with mean FOG score 0.681. In comparison with HC, PD participants (median of 4.7 months from diagnosis) were relatively younger, taller, had proportionally more males, lower balance confidence (ABC), and poorer cognition (MMSE).

**Table 1:** Demographic and clinical characteristics; M: Male; F: Female; BMI: Body mass index; MMSE: Mini-mental state examination; ABC: Activities specific balance confidence scale; UPDRS: Unified Parkinson’s disease rating scale; PIGD: Postural instability and gait disorder phenotype; ID: Indeterminate phenotype; TD: Tremor dominant phenotype; t(df): t-value at degree of freedom; p showing the statistical difference between PD and HC. In bold significant p values (p < 0.05).

Characteristics	HC (n = 184) Mean ± SD	PD (n = 119) Mean ± SD	t(df)	p
M/F (n)	78/106	79/40	—	**<0.001**
Age (year)	69.974 ± 7.711	66.898 ± 10.488	t(199.23) = 2.75	**0.006**
Height (m)	1.675 ± 0.097	1.696 ± 0.083	t(278.05) = −2.02	**0.045**
Weight (Kg)	76.544 ± 14.691	78.678 ± 15.115	t(301) = −1.22	0.223
BMI (Kg/m^2^)	27.169 ± 3.913	27.233 ± 4.396	t(300) = −0.13	0.896
MMSE (0–30)	29.29 ± 1.019	28.66 ± 1.304	t(301) = 4.69	**<0.001**
ABCs (0–100%)	91.816 ± 10.902	82.597 ± 18.985	t(301) = 5.36	**<0.001**
Levodopa equivalent daily dose (LEDD, mg/day)	—	175.893 ± 143.724	—	—
Freezing of gait score (FOG)	—	0.681 ± 2.718	—	—
Hoehn and Yahr - Median	—	2	—	—
Hoehn and Yahr (n) - HY I	—	28	—	—
HY II	—	70	—	—
HY III	—	21	—	—
Time from Clinical Diagnosis (months)	—	6.23 ± 4.89	—	—
MDS-UPDRS III – Item 3.10	—	0.571 ± 0.671	—	—
MDS-UPDRS III – overall	—	25.37 ± 10.399	—	—
MDS-UPDRS III for HY I	—	16.82 ± 5.604	—	—
MDS-UPDRS III for HY II	—	27.49 ± 10.61	—	—
MDS-UPDRS III for HY III	—	29.71 ± 8.307	—	—
Motor Phenotype (n) - PIGD	—	55	—	—
ID	—	11	—	—
TD	—	33	—	—

### Input features as training data

**Figure 3:**
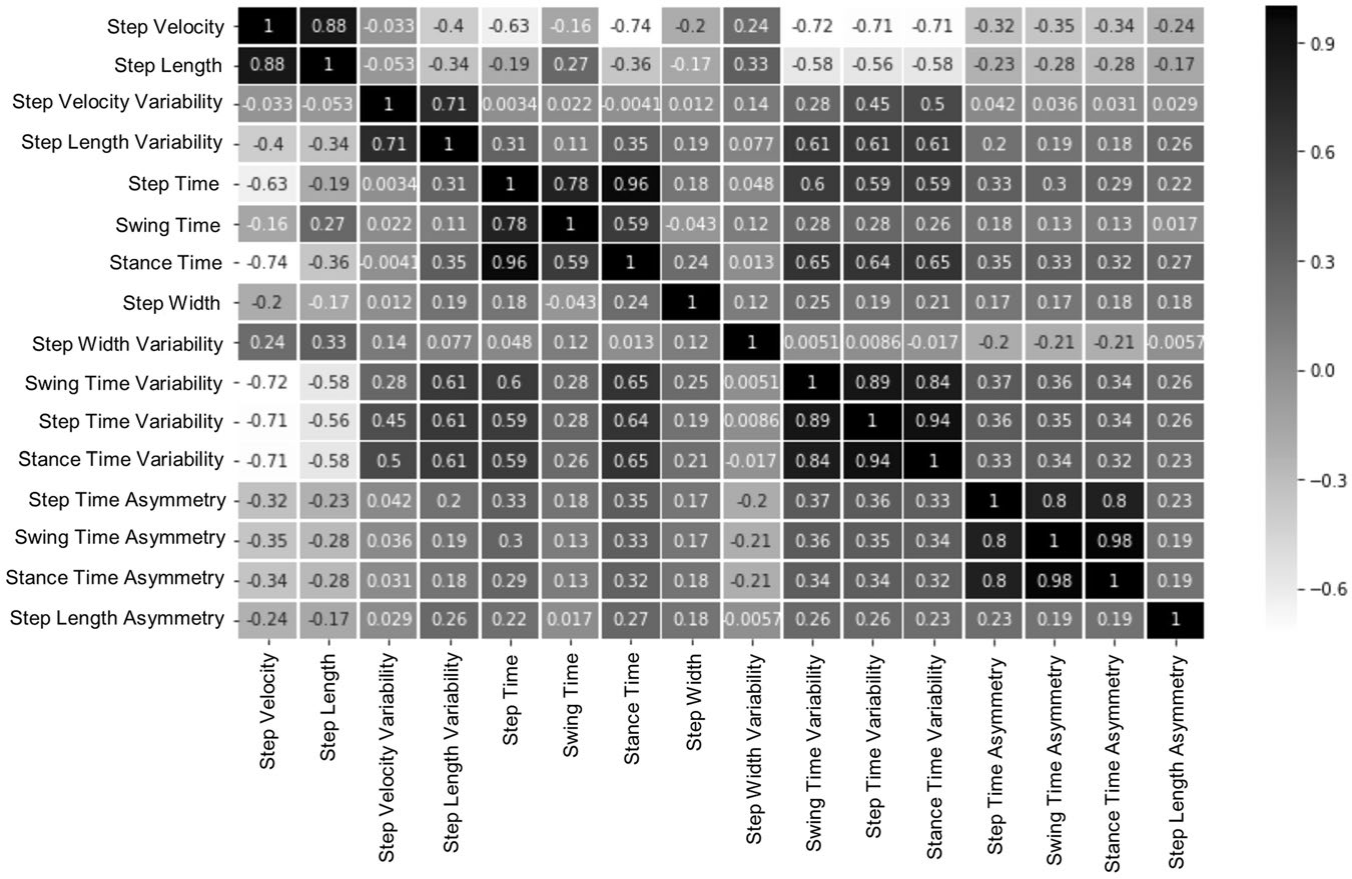
Heat map showing the correlation among the 16 gait characteristics.

From Table 2, all gait domains differed significantly between groups and 13 out of 16 gait characteristics were significantly impaired in PD. When looking at the association between the gait characteristics in Fig. 3 we found a number of highly correlated characteristics. As these gait characteristics are not independent due to their high correlation, it was important to find the optimal combination of gait characteristics for classification of PD to avoid redundancy.

**Table 2:** Significant difference between PD and HC; AUC: Area under the curve; p showing the statistical difference between PD and HC. In bold significant p values (p < 0.05).

Gait Model Domain	Gait Characteristics	HC (n = 184) Mean ± SD	PD (n = 119) Mean ± SD	p	AUC
Pace	Step Velocity (m/s)	1.264 ± 0.192	1.125 ± 0.213	**<0.001**	0.695
	Step Length (m)	0.672 ± 0.083	0.623 ± 0.101	**<0.001**	0.655
	Swing Time Variability (s)	0.015 ± 0.005	0.018 ± 0.006	**<0.001**	0.636
Rhythm	Step Time (s)	0.537 ± 0.047	0.560 ± 0.049	**<0.001**	0.628
	Swing Time (s)	0.387 ± 0.030	0.392 ± 0.033	0.170	0.541
	Stance Time (s)	0.688 ± 0.072	0.728 ± 0.077	**<0.001**	0.646
Variability	Step Velocity Variability (m/s)	0.053 ± 0.013	0.054 ± 0.017	0.576	0.510
	Step Length Variability (m)	0.020 ± 0.006	0.023 ± 0.008	**0.001**	0.612
	Step Time Variability (s)	0.016 ± 0.006	0.019 ± 0.006	**0.001**	0.624
	Stance Time Variability (s)	0.019 ± 0.008	0.023 ± 0.009	**0.001**	0.611
Asymmetry	Step Time Asymmetry (s)	0.011 ± 0.010	0.023 ± 0.028	**<0.001**	0.654
	Swing Time Asymmetry (s)	0.009 ± 0.009	0.017 ± 0.020	**<0.001**	0.675
	Stance Time Asymmetry (s)	0.008 ± 0.009	0.017 ± 0.019	**<0.001**	0.675
Postural Control	Step Width (m)	0.089 ± 0.025	0.093 ± 0.031	0.348	0.527
	Step Width Variability (m)	0.022 ± 0.005	0.019 ± 0.006	**<0.001**	0.682
	Step Length Asymmetry (m)	0.020 ± 0.017	0.026 ± 0.022	**0.024**	0.568

### Selected machine learning models

All 16 gait characteristics (Table 2) were used for classification modelling. We adopted a comprehensive approach to select the optimal ML model. Table 3 shows spot checking results based on the whole dataset with 10-fold cross validation under default hyper-parameters of models. Baseline accuracy based on a zero rule algorithm was 60.72%. All linear models performed almost the same with around 80% classification accuracy between PDs and HCs. In other models, SVM with radial basis function (RBF) kernel accuracy was about 84% and in ensemble models such as RF and GBC, showed 86% and 85% accuracy respectively. Selection of the classification models was refined to five models (LR, LDA, SVM-RBF, RF, and GBC) following spot checking of 12 models based on model domain and were subsequently tested using the test harness described in Fig. 2 using training and testing data separately. Results are presented in Table 4.

**Table 3:** Spot checking results of the models; RBF: Radial basis function.

Model	Accuracy % Mean ± SD
Logistic Regression (LR)	79.9 ± 4.6
Linear Discriminant Analysis (LDA)	79.6 ± 7.9
K-Nearest Neighbour (KNN)	76.1 ± 5.9
Classification and Regression Tree (CART)	73.4 ± 4.1
Naïve Bayes (NB)	78.3 ± 7.4
Support Vector Machine (SVM-RBF)	83.9 ± 7.1
Random Forest (RF)	86.0 ± 8.4
Bagged Decision Tree (BDT)	80.1 ± 8.7
Extra Tree Classifier (ETC)	83.4 ± 7.1
Adaboost Classifier (AC)	82.1 ± 7.8
Gradient Boosting Classifier (GBC)	84.9 ± 7.6
Voting Classifier (LDA, Naïve Bayes, SVM)	78.7 ± 6.4

**Table 4:** Checking model performance on training and testing data; SE: Sensitivity, SP: Specificity; RBF: Radial basis function.

Refined Models	Training Accuracy % Mean ± SD	Testing Accuracy % Mean (SE, SP)
Random Forest (RF)	87.94 ± 6.88	87.14 (94, 79)
Gradient Boosting Classifier (GBC)	85.34 ± 7.38	84.28 (89, 79)
Support Vector Machine (SVM-RBF)	83.37 ± 7.35	81.42 (78, 85)
Linear Discriminant Analysis (LDA)	75.89 ± 9.56	82.85 (63, 85)
Logistic Regression (LR)	79.65 ± 11.22	82.85 (68, 79)

Training accuracy (based on the 10-fold cross-validation) and testing accuracy were almost similar for RF. For GBC and SVM there was a slight decrease in the testing accuracy to a maximum of 2%. On the other hand, both linear models (LDA and LR) had lower training accuracy but similar testing accuracy compared to other models. For further analysis, only three models were selected from these five models. Each model was selected from a different domain, such as RF from ensemble or tree based approach, SVM due to kernel techniques, and LR due to linear models were selected for further fine-tuning to get the optimal results. During fine-tuning on training data, the hyper-parameters (such as number of trees and regularization coefficients) were determined by cross validation.

### Selected gait characteristics for optimal results

**Figure 4:**
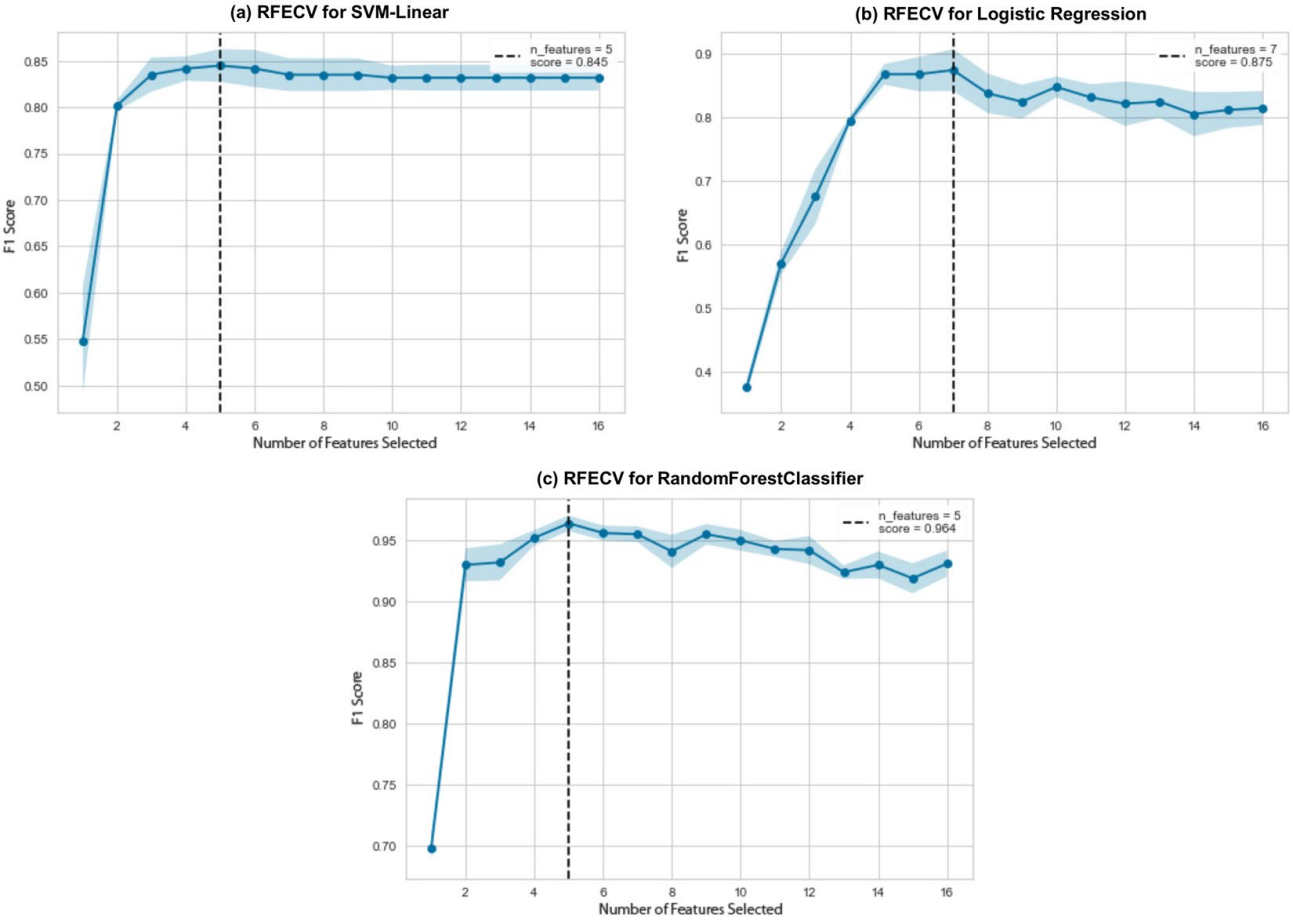
Selection of optimal number of gait characteristics with (**a**) support vector machine, (**b**) logistic regression, (**c**) random forest.

Based on the RFE technique in Fig. 4(a–c), the optimal performance in RF and SVM was achieved with five gait characteristics. However for LR, the optimal performance was achieved with seven characteristics, and after five characteristics the performance decreased drastically. From this analysis, it was clear that with five gait characteristics optimal performance can be achieved. Performance evaluation of the models with RFE is performed with 10-fold cross-validation (RFECV). The F1 score was used for this purpose to find the balance between precision and recall as shown in the Fig. 4(a–c). The RF has a classification score of 96.4% with five gait characteristics. Similarly, for SVM, the best F1 score was 84.5% with five gait features. However, for LR the score was 87.5% with seven gait characteristics.

**Figure 5:**
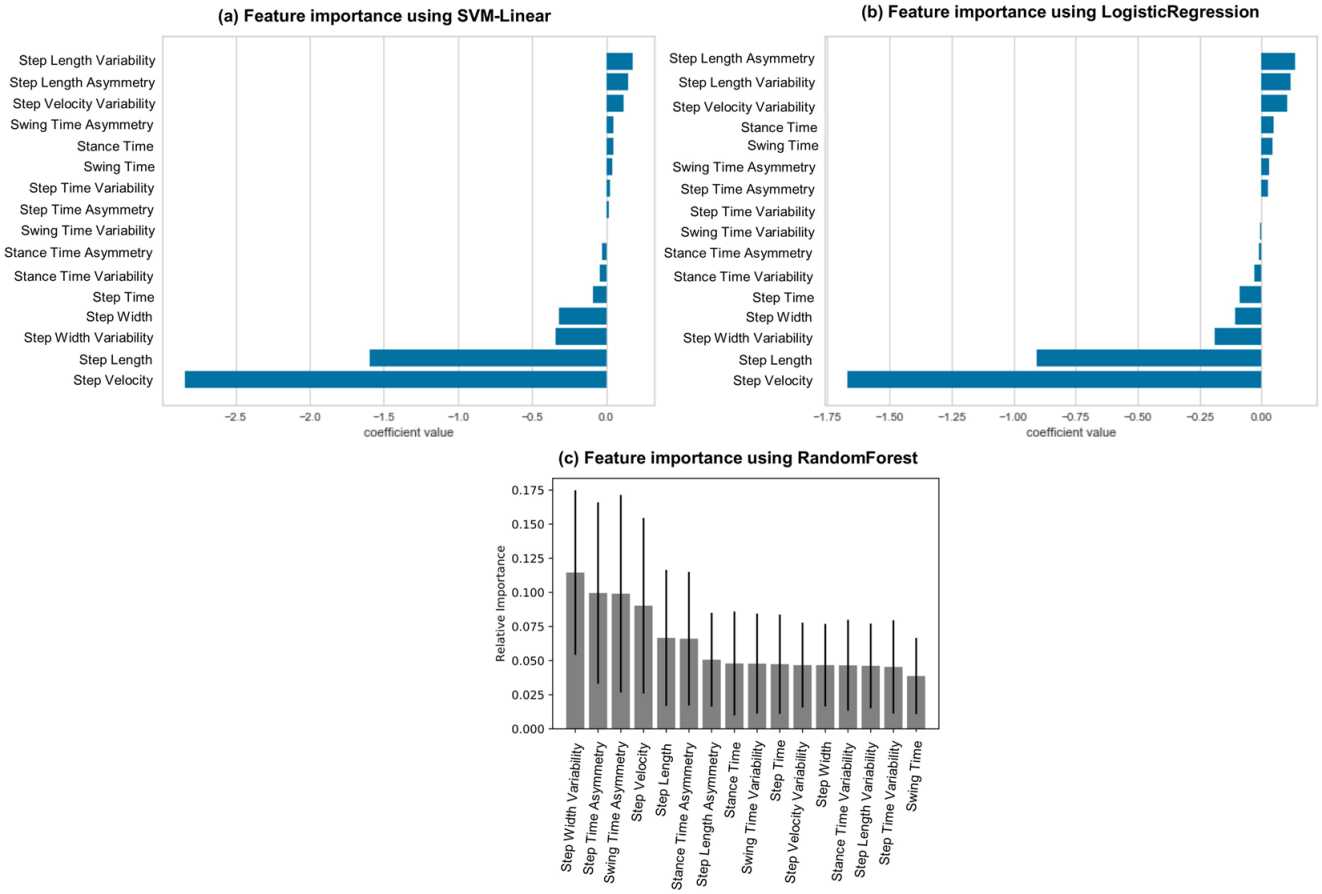
Feature selection with (**a**) support vector machine, (**b**) logistic regression, (**c**) random forest.

Figure 5(a–c) show the contribution of each gait characteristic in the classification model. The six common gait characteristics among the top 10 were mean step velocity, step length, step time, stance time; step width variability; and step length asymmetry. The ML models trained with different gait characteristics (top five selected with each ML model, top 10 selected with each ML model, common among 10 in all models, and top five selected with linear-SVM) were evaluated on testing data to identify the optimal combination.

The testing results of the RF, SVM-RBF, and LR are presented in Table 5 with the F1 score representing the training results based on RFE technique. Overall, RF performed better than SVM-RBF and LR. From a total of 16 gait features, the top ten gait characteristics selected by each model gave good testing classification accuracy. RF showed 94.28% accuracy with 100% sensitivity and 89% specificity, followed by the LR which had 82.85% accuracy with 71% sensitivity and 89% specificity; and SVM-RBF showed 81.92% accuracy with 71% sensitivity and 89% specificity. With the common features selected by all the models, RF performance decreased slightly, SVM-RBF classification accuracy increased, and for LR it remained almost the same. With the top five gait characteristics, we observed the same classification accuracy for RF as with the top ten features. However, for the SVM-RBF, the accuracy increased to 85.71% by reducing the feature set. A similar case was with LR, the accuracy increased to 84.28% by reducing the feature set. Further, we also observed that, if we feed five gait characteristics selected with linear-SVM-RFE, then all the models gave optimal performance. The final optimal performance from RF was 97.14% classification accuracy with 100% sensitivity and 94% specificity. For SVM-RBF it was 85.71% accuracy with 79% sensitivity and 94% specificity. Similar for LR it was 84.99% accuracy with 76% sensitivity and 94% specificity. In addition, the training accuracy with 10-fold cross validation is evaluated in terms of the F1 score to have single measures to check the performance of the model. RF has the highest F1 score of 96.4% followed by LR of 87.5% and SVM of 84.5%.

**Table 5:** Optimal classification accuracy on testing and training data; GC: Gait characteristics; SE: Sensitivity, SP: Specificity; RFE: Recursive features elimination technique; RF: Random forest; SVM-RBF: Support vector machine with radial basis function kernel; LR: Logistic regression.

Models	Top 10 GC – Test Accuracy% Mean (SE, SP)	Common GC – Test Accuracy% Mean (SE, SP)	Top 5 GC – Test Accuracy% Mean (SE, SP)	SVM top 5 GC – Test Accuracy % Mean (SE, SP)	Training F1 Score in RFE on optimal number of GC %
RF	94.28 (100, 89)	91.42 (94,89)	94.28 (100, 89)	97.14 (100, 94)	96.4
SVM-RBF	81.92 (71, 89)	83.67 (72, 94)	85.71 (79, 94)	85.71 (79, 94)	84.5
LR	82.85 (74, 94)	82.54 (79, 90)	84.28 (76, 92)	84.99 (76, 94)	87.5

## Discussion

Based on the best of our knowledge, this is the largest classification study in PD using a comprehensive approach to determine the optimal ML model and spatiotemporal gait features. Gait features were selected according to a validated model of gait in PD in participants with relatively early disease^6^. We were able to identify both the optimal ML model and combination of gait characteristics for classification of PD. We found that by using only five gait characteristics from three independent gait domains (pace, postural control, and variability) selected by RFE-SVM we were able to achieve optimal PD classification. The highest testing classification accuracy of 97% with 100% sensitivity and 94% specificity was achieved with RF.

Sixteen gait characteristics from five domains (pace, rhythm, variability, asymmetry and postural control) were used as input features for classification (see Supplementary Fig. S1 for data distribution). Pace, rhythm, variability, asymmetry and postural control characteristics differed significantly between groups. PD walked at a slower pace (slower and with shorter steps) and rhythm, with impaired postural control (higher step length asymmetry and lower step width variability) and with a more variable and asymmetric gait pattern compared to HC. This is in line with previous research on gait impairment in PD^6,11,15,20,50^.

Only a few studies reported the feature selection processes used to identify the importance of gait characteristics in ML modelling with walking speed (step velocity), step/stride length, stride time, and step time asymmetry identified as important features for classification of PD^3,21,22^. There are some notable exclusions, as none of these studies included gait related postural control features in their models (e.g. step width and step width variability) which have been shown to be important and sensitive indicators of gait impairment in PD^6,15^. In this study, we first presented a feature selection phase and we identified step velocity, step length, step width variability, step width and step length variability as important characteristics to classify PD. Selection of step velocity and step length is in line with previous studies^3,21,22^. We were surprised to see that both were included because of the high correlation between these characteristics. Adopting a data driven approach however indicates that the spatial component of step velocity (e.g. step length) retains additional information that is an important “independent,” explaining its selection. Conversely it seems that step time does not contribute additional information to step velocity and hence was not selected (“important”) as a feature in the top five. Step width and step width variability (standard deviation of step widths^51^) had no or low correlation with other gait characteristics and were highly relevant in the selection process, so we suggest that these are variables to be included in future classification studies. Only one study used step width in machine learning and did not report its importance; the accuracy achieved in this study was 93%^20^. From a clinical perspective, it also makes sense that gait related postural control features are important for classification, based on evidence that postural control is a specific biomarker for neurodegenerative diseases and in particular for PD^6,15^.

Selection of the ML models for PD classification was based on an extensive ML framework. First, a comprehensive approach was utilized to include models used in previous studies^3,20,22,32,33,42^ and models such as the LR, LDA, KNN, CART, NB, SVM, RF, BDT, ETC, AC, GBC, and the voting method were therefore implemented. Previously, LR was used with eight feet force sensor data for classification between PD and HC^32^. LDA was trained on the statistical features extracted from two Shimmer sensors and obtained a classification accuracy of 82%^42^. Similarly, KNN^11,20,22,43^, SVM^11,20,22,42^ with linear^32^ and non-linear kernels^3,21,30^, CART^22^, NB^20,22^, RF^11,20,32,44^, and majority voting^22^ were used to get reasonable classification accuracy. Based on spot-checking in our study, we found ensemble models such RF, GBC, BDT, ETC and AC performed better with an overall classification accuracy of 86%. The non-linear SVM-RBF model gave classification accuracy of 84%. From linear models, LR and LDA gave similar classification accuracy of 80%. Based on these results in the initial model selection phase, five classification models RF, GBC, SVM, LDA, and LR were therefore selected.

The deployment of the ML model in real world practice is still unknown due to issues with generalizability. In order to test the robustness of ML models, independent/external datasets which have not been used in the training of the model should be used to validate model performance. To our knowledge, there are only two studies that used independent datasets for checking the performance of the proposed models^42,43^. Their classification accuracy ranged between 81 to 85.71%. However, most studies used 10-fold cross-validation methods, due to their small sample sizes. As a consequence generalizability of the models remains unclear. To date the largest study (PD:156, HC:424)^43^ reported the classification accuracy of 85.71% but did not include the sensitivity and specificity of their models. Using a smaller dataset (DP:12, HC:20)^21^, accuracy was up to 100% with sensitivity ranging between 9.03–100% and specificity ranging between 86.7–100%, however model overfitting can bias the results and provide an unstable performance when other evaluation metrics are included (e.g., F1 score). To overcome these limitations we proposed a test harness in the ML framework where our five selected models were trained and tested separately using independent data. Different split ratios for training and testing (70/30%, 80/20%, and 90/10%) were used, due to similar results and to have more data for training, 90% data for training and 10% for independent testing were used in final analysis. Overall training accuracy of 76–88% based on 10-fold cross-validation was achieved and a similar accuracy of 81–87% was achieved on the test data. Further hyper-parameter tuning and feature reduction in RF, SVM, and LR gave the highest test classification in a range of 81–97.14% with 71–100% sensitivity and specificity of 89–94%. In this context we report 100% sensitivity to indicate that models were able to classify all people with PD correctly. With 94% specificity, some of the HC were classified as the PD. In the real world, high sensitivity compared to specificity may be optimal to avoid misdiagnosis in initial screening. We also reported another commonly used metric (F1 score) which was between 85–96%. All these results are higher or comparable to the previous studies^3,11,20–22,30,32,33,42,44^.

Motivation of using ML for feature selection was to extract the discriminatory features while suppressing the redundant features. Even though the data between groups was overlapping, based on some extracted features, we can see ML models effectively classify PD and HC groups. In order to select the optimal gait characteristics for the model we chose the recursive feature elimination (RFE) wrapper based method which has advantages over other filter based methods^52^. This is an iterative method where features are removed one by one rather than in combination. As the ranking of features is based on a single gait characteristic, this technique will have no effect on methods using correlations^49^. The space dimensionality of the gait characteristics is reduced with RFE and the least related gait characteristics are removed one by one without having an effect on the training error.

ML models can also give different importance weights to features depending upon the nature of the models. Based on analysis the top five gait characteristics were enough for optimal PD classification. These characteristics belong to pace, variability and postural control followed by asymmetry and rhythm domains of gait model^15^. In this study, RF gave a relatively high importance to step width variability, step time asymmetry, swing time asymmetry, step velocity, and step length. RFE also gave similar results with SVM and LR models where the same four features were selected (step velocity, step length, step width variability, step width), with a difference on the 5^th^ gait characteristic (step length variability with SVM and step length asymmetry with LR). The features selected with SVM gave the highest classification accuracy and this model is in line with previous work^11^.

It’s possible that, some of the results may be influenced by more severe PD (HY III), despite the fact that subjects had gait assessment with a median of 4.7 months from clinical diagnosis with relatively low doses of dopaminergic medication. To check the original results, analysis was re-run by removing the 21 subjects at HY III. Based on analysis, classification performance ranged in between 75.75–96.11% with 76–95% sensitivity and 78–95% specificity. RF gave best classification performance on the features selected with RFE-SVM. The same first four gait characteristics (step velocity, step length, step width variability, step width) were selected and the same model (RF) gave the optimal performance. The performance of the models was comparable to whole data set including HY III, with slightly less sensitivity and high specificity. Due to the heterogeneous nature of PD, even in early disease, there will be a range of motoric and cognitive abilities. The inclusion of these participants ensures that our dataset is generalizable to those seen in clinics. Therefore we used the entire dataset for the final analysis.

ML methods appear to be more sensitive to overall variability in the data compared to simple statistical methods, which is important to understand for classification of early PD. In classification studies for healthcare application, the addition of a feature selection and reduction phase using ML plays an important role to tackle the problem of model overfitting, limiting the impact of the noise in the data during the classification phase. Further, feature reduction can help to improve model accuracy, as seen in our study where the accuracy of the models increased to 97% when redundant features were removed. This also reduces training time, augmenting the overall ML performance and implementation. From a clinical perspective, classification with ML techniques can help clinicians to use ML as a tool to support diagnosis of PD and provide an explanation for informed decision making. Our findings also help pave the way to enhance the utility of ML for clinicians.

There are some limitations in this study. One model of gait including specific gait characteristics was included in this work, and whilst comprehensive, in the future other reported models and outcomes should be considered to identify the best measure (or combination of measures) for classification of early PD. Due to the large cohort size, there was an imbalance between gender and a statistical difference between the gender, age, and height. This is reasonable for achieving the model generalizability on a diverse dataset, however, classification results may improve with a more homogeneous dataset. In this early cohort, HY III PD were included with very low FOG score, LEDD intake was relatively low, and the MDS-UPDRS III score was low in support of a mildly affected group. Due to the heterogeneous nature of PD, inclusion of these participants ensures that our dataset is generalizable to those seen in clinics. These were early stage PD without post-mortem confirmation, therefore it is possible that a small number may have an alternative diagnosis. However, participants continue to be followed up every 18 months with consideration of alternative diagnoses given at that time. This was not a de novo group, and thus may limit the generalisability, although our cohort reflects clinical practice. In this study, gait characteristics were derived from an instrumented mat (GAITRite); however, a similar analysis should also be performed with wearable sensors to investigate the contribution of the characteristics in ML classification models. Only single-task gait characteristics were analysed in this study, in future the contribution of the dual-task gait characteristics in PD classification models will also be investigated. The findings in this study are based on the test results (10-fold cross-validation and on 10% testing data) on our cohort dataset (in a controlled setting), which may not generalise well to other cohorts. In future, we aim to evaluate our findings on much larger datasets (with diverse cohorts) in more naturalistic environments.

## Conclusion

In this study, comprehensive ML approaches were used to identify suitable models and the most important combination of spatial-temporal gait characteristics for classification of early PD. The best classification models for our dataset were RF, SVM, and LR. Following feature selection, model performance improved by 10%. RF gave the highest testing classification accuracy of 97% with features selected with RFE-SVM such as mean step velocity, mean step length, step width variability, mean step width, and step length variability. These features not only give better results but pave the way for an enhanced understanding of ML for clinicians. The findings are the first step to demonstrate the potential of ML as a complementary tool to support clinical practice, however further external validation is needed to confirm these findings.

## Supplementary information

Figure S1

## Data Availability

All the digital gait characteristics are presented in the Table 2 in the manuscript. Also the distribution of the data is shown through violin plots in Supplementary Fig. S1. Due to data privacy and sharing agreement, the complete dataset is not publically available. However, it can be available upon reasonable request from corresponding author (lynn.rochester@ncl.ac.uk).
