# Supplementary material for: Selecting Clinically Relevant Gait Characteristics for Classification of Early Parkinson’s Disease: A Comprehensive Machine Learning Approach
Source: Sci Rep. 2019 Nov 21;9:17269. doi: 10.1038/s41598-019-53656-7 (PMC6872822; doi:10.1038/s41598-019-53656-7)
Supplement: Supplementary file 1 — Figure S1 [file 41598_2019_53656_MOESM1_ESM.docx]

**Selecting Clinically Relevant Gait Characteristics for Classification of Early Parkinson’s Disease: A Comprehensive Machine Learning Approach**

Rana Zia Ur Rehman^1^, Silvia Del Din^1^, Yu Guan^2^, Alison J. Yarnall^1, 4^, Jian Qing Shi^3^ and Lynn Rochester^1, 4^

^1^Institute of Neuroscience/Institute for Ageing, Newcastle University, Newcastle Upon Tyne NE4 5PL, U.K.

^2^School of Computing, Newcastle University, Newcastle Upon Tyne, NE4 5TG, U.K.

^3^School of Mathematics, Statistics, and Physics, Newcastle University, Newcastle Upon Tyne NE1 7RU, U.K.

^4^The Newcastle upon Tyne Hospitals NHS Foundation Trust, Newcastle Upon Tyne NE7 7DN, UK

Rana Zia Ur Rehman: [rana.zia-ur-rehman@ncl.ac.uk](mailto:rana.zia-ur-rehman@ncl.ac.uk)

Silvia Del Din: [silvia.del-din@ncl.ac.uk](mailto:silvia.del-din@ncl.ac.uk)

Yu Guan: [yu.guan@ncl.ac.uk](mailto:yu.guan@ncl.ac.uk)

Alison J. Yarnall: [alison.yarnall@ncl.ac.uk](mailto:alison.yarnall@ncl.ac.uk)

Jian Qing Shi: jian.shi@ncl.ac.uk

Lynn Rochester: [lynn.rochester@ncl.ac.uk](mailto:lynn.rochester@ncl.ac.uk)

^*^Corresponding author: Prof. Lynn Rochester

Phone: +44 (0) 191 208 1291

Fax: +44 (0) 191 208 1251

**Supplementary Material**


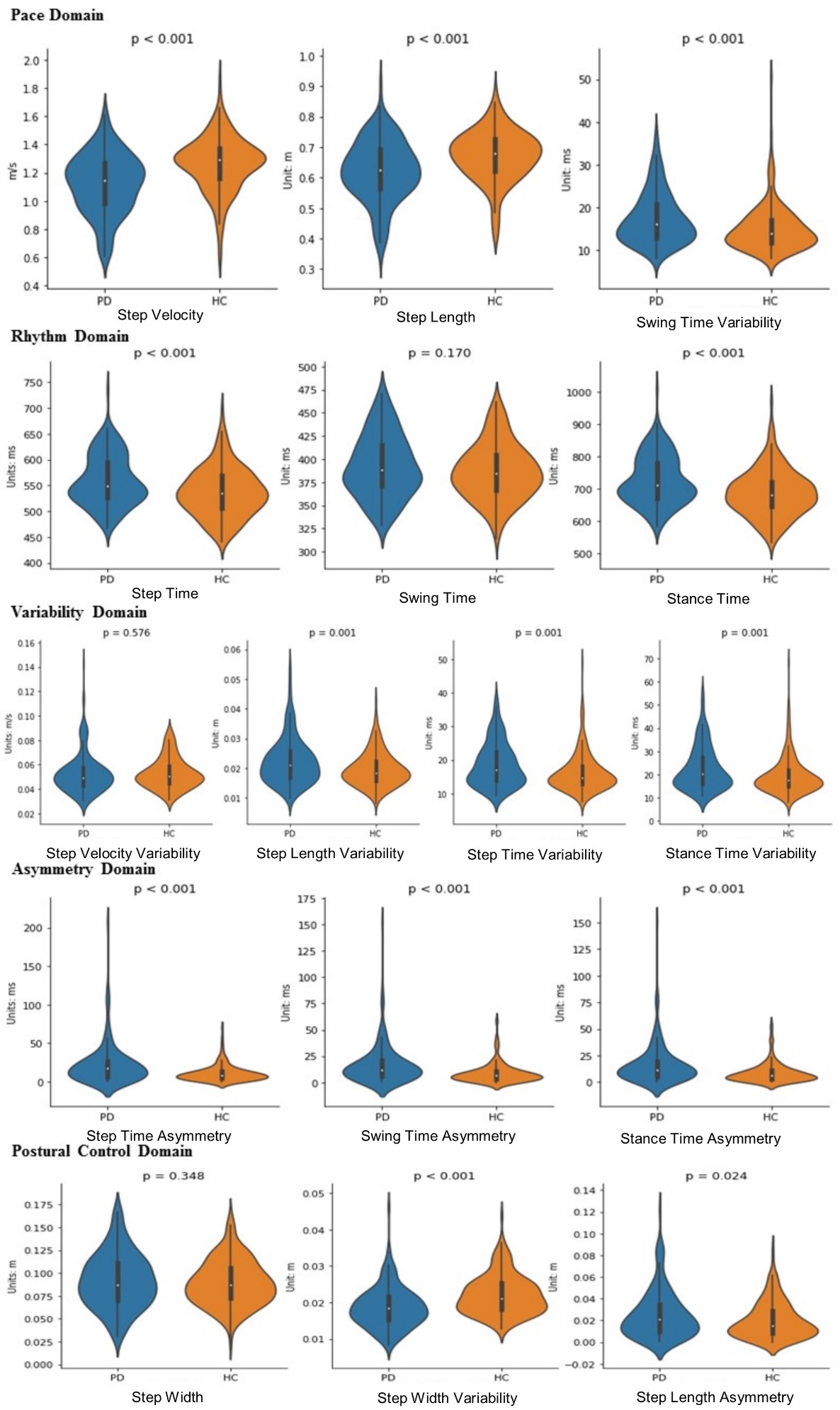


Figure S1: Violin plots for 5 domain of gait model explaining the distribution of the gait characteristics
